# Supplementary figures and images for: A Versatile High Throughput Screening Platform for Plant Metabolic Engineering Highlights the Major Role of ABI3 in Lipid Metabolism Regulation
Source: Front Plant Sci. 2020 Mar 17;11:288. doi: 10.3389/fpls.2020.00288 (PMC7090168; doi:10.3389/fpls.2020.00288)

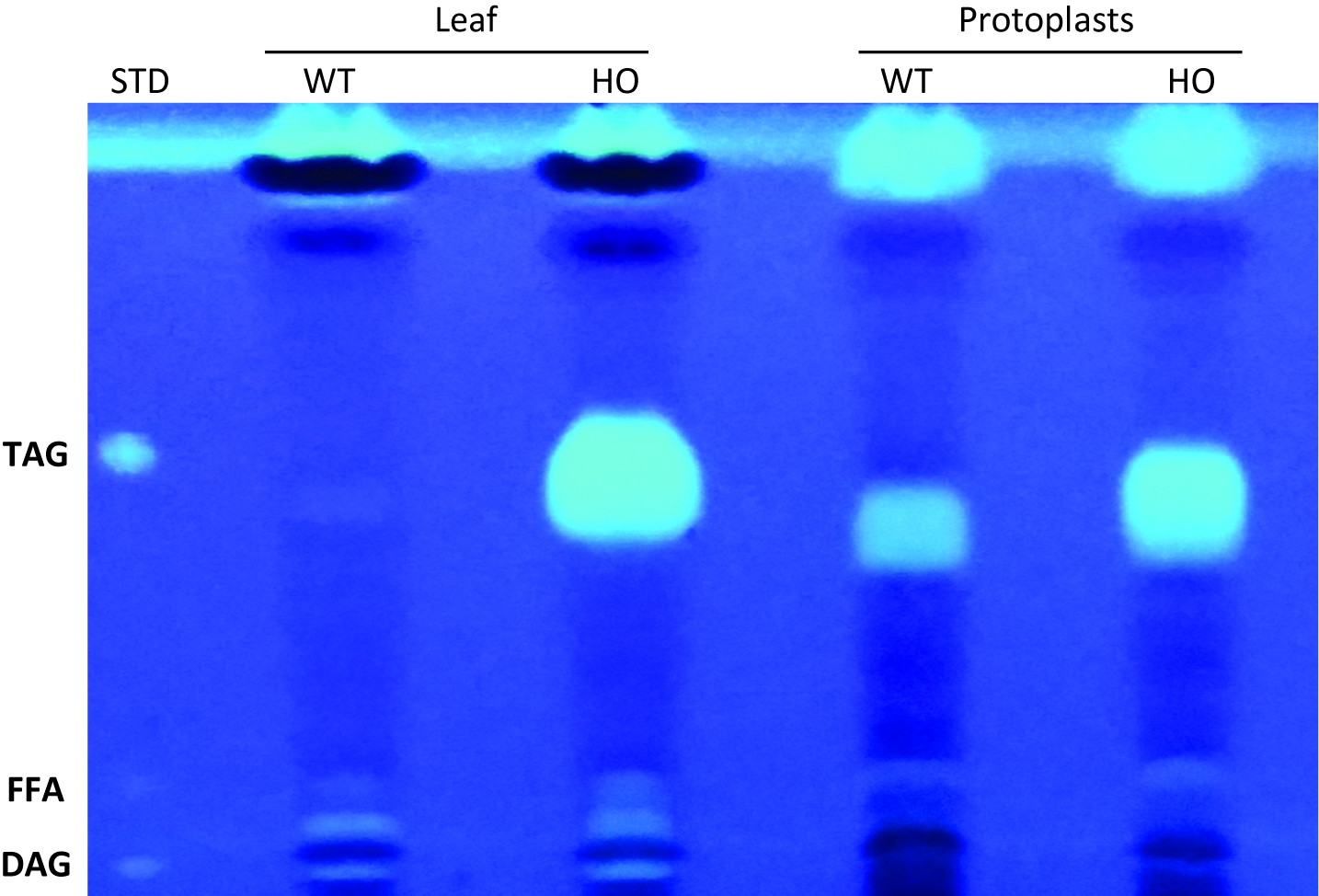

Supplement: FIGURE S1 — Repeat of thin layer chromatography plate presented in Figure 2. Thin layer chromatography plate separation of total fatty acids (TFA) extracted from leaves or from isolated protoplasts of WT or HO lines. STD stands for standards run in parallel of the samples to validate the location of the lipid. [file Image_1.TIF]
