# Supplementary material for: A Versatile High Throughput Screening Platform for Plant Metabolic Engineering Highlights the Major Role of ABI3 in Lipid Metabolism Regulation
Source: Front Plant Sci. 2020 Mar 17;11:288. doi: 10.3389/fpls.2020.00288 (PMC7090168; doi:10.3389/fpls.2020.00288)
Supplement: TABLE S1 — Raw data for lipid quantifications presented in Figures 2, 3. Note: TAG/TFA content in tobacco leaf is presented in % of fresh weight leaf and in protoplasts in μg/1 M cells. [file Table_1.DOCX]

Supplemental Table 1:

Raw data for lipid quantifications presented in figure 2

Raw data for lipid quantifications presented in figure 3

Note: TAG/TFA content in tobacco leaf is presented in % of fresh weight leaf and in protoplasts in µg/1M cells.
